# 6-Bromoindirubin-3′-Oxime (6BIO) Suppresses the mTOR Pathway, Promotes Autophagy, and Exerts Anti-aging Effects in Rodent Liver

**DOI:** 10.3389/fphar.2019.00320

**Published:** 2019-04-10

**Authors:** Donghao Guo, Yun Shen, Wei Li, Qinjie Li, Yu Zhao, Chenhao Pan, Bi Chen, Yuan Zhong, Ya Miao

**Affiliations:** ^1^ Department of Geriatrics, Shanghai Jiao Tong University Affiliated Sixth People’s Hospital, Shanghai, China; ^2^ Department of Cardiology, Shanghai Jiao Tong University Affiliated Sixth People’s Hospital, Shanghai, China; ^3^ Department of Orthopedics, Shanghai Jiao Tong University Affiliated Sixth People’s Hospital, Shanghai, China

**Keywords:** 6-bromoindirubin-3′-oxime, aging, autophagy, mammalian target of rapamycin, glycogen synthase kinase-3β

## Abstract

Liver aging is associated with age-related histopathological and functional changes that significantly enhance the risk of numerous diseases or disorders developing in elderly populations. 6-Bromoindirubin-3′-oxime (6BIO), a potent inhibitor of glycogen synthase kinase-3 (GSK-3), has been implicated in various age-related diseases and processes, such as tumorigenesis, neurodegeneration, and diabetes. Recent studies have also revealed that 6BIO increases autophagy in yeast, mammalian cell lines, and dopaminergic neurons, which is one of the classical mechanisms strongly associated with liver aging. However, the impact or the mechanism of action of 6BIO in liver remains entirely unknown. Here, we find that 6BIO reduces oxidative stress, improves lipid metabolism, enhances autophagy, and significantly retards liver aging *via* modulating the GSK-3β pathway and mTOR pathway. Our findings suggest that 6BIO could be a potential agent to protect the liver in the field of anti-aging pharmacology.

## Introduction

Aging is broadly defined as the time-dependent functional decline of a living organism, usually accompanied by the age-related gradual accumulation of damaged biomolecules, which eventually results in the disruption of cellular homeodynamics. This deterioration is the primary risk factor for major human pathologies, including neurodegenerative diseases cancer and diabetes (Lopez-Otin et al., 2013). The development of pharmacological intervention in age-related functional decline and pathological changes has attracted much attention in the field of biology (Kennedy and Pennypacker, 2014; Vaiserman et al., 2016).

GSK-3 was first identified by its role in glycogen synthase phosphorylation (Embi et al., 1980) and later reported as a key protein that may function as an intermediate in inflammation and metabolism (Orellana et al., 2015). 6-Bromoindirubin-3′-oxime (6BIO) is a selective inhibitor of glycogen synthase kinase-3α/β (GSK-3α/β) derived from Tyrian purple indirubin (Zhao et al., 2017). It is reported as a promising novel agent for therapeutic intervention in several age-related diseases, including diabetes, neurodegenerative disorders, leukemia, and cancer (Avrahami et al., 2013; Shen et al., 2015; Zhang et al., 2017; Li et al., 2018; Liu et al., 2018). It also suppresses the cellular senescence-related accumulation of damaged biomolecules and modulates cellular processes associated with aging, such as inflammation, oxidative stress, cellular viability, proliferation, and apoptosis (Nicolaou et al., 2012; Tsakiri et al., 2017; Zhang et al., 2017).

Recent studies have revealed that 6BIO is also a potent autophagy modulator. Accordingly, 6BIO reduces the oxidative load and upregulates autophagy-related protein Beclin1 in human diploid skin fibroblasts (Sklirou et al., 2017). Another study found that 6BIO induced autophagy in dopaminergic neurons of mice midbrain in order to clear toxic protein aggregates in a preclinical model of Parkinson disease, the second most common neurodegenerative disorder, and a type of age-related disease. This research also implies that 6BIO diminishes phosphorylation of ribosomal protein S6 kinase B1 (RPS6KB1) and eukaryotic translation initiation factor 4E-binding protein 1 (EIF4EBP1), which are downstream targets of the mammalian target of rapamycin (mTOR) (Suresh et al., 2017).

However, especially in *in vivo* models, relevant studies about 6BIO are mainly confined to the area of neurology, immunology, and oncology (Braig et al., 2013; Klamer et al., 2013; Suresh et al., 2017; Zhao et al., 2017). Whether 6BIO can suppress liver aging, and the mechanisms underlying its probable anti-aging effects, remains unclear. Considering that lipid metabolism is closely related to liver function and that autophagy, as well as inflammation and oxidative stress, is a vital factor in liver aging (Martinez-Cisuelo et al., 2016), we assume that 6BIO, which affects lipid metabolism and modulates autophagy, inflammation, and oxidative stress, may also play a pivotal role in liver aging *via* autophagic pathways.

Therefore, to determine the role of 6BIO in liver aging, we evaluated the effect of 6BIO on aging characteristics and age-related hepatic changes. We also investigated the possible molecular mechanisms involved in the geroprotective effect of 6BIO. Our results suggested that 6BIO treatment significantly ameliorates age-related changes, including reducing oxidative stress, improving lipid metabolism, and enhancing autophagy through the mTOR and GSK-3β pathways.

## Materials and Methods

### Animals

Male young (2-month-old) and aged (18-month-old) mice were purchased from the Experimental Animal Center of the Chinese People’s Liberation Army Fourth Military Medical University and were maintained on a 12:12 h light/dark cycle with lights on at 8:00 am. All mice had *ad libitum* access to water and food. The experiment was performed on four different groups, with eight mice per group: a young control group, an aged control group, a 6BIO treatment group, and a rapamycin treatment group. The rapamycin treatment was used as the positive control because rapamycin is reported to ameliorate liver aging by inducing mTOR-regulated autophagy (Ehninger et al., 2014). Young male mice were used in the young control group, and aged male mice were used in the other three groups. To test the anti-aging effect of 6BIO and rapamycin, the mice were injected with 6BIO (10 mg/kg) and rapamycin (4 mg/kg) in 10 ml/kg of saline intraperitoneally every day for 2 weeks (Siegmund et al., 2017; Suresh et al., 2017). The young control group and the aged control group received the same volume (10 ml/kg) of 0.9% NaCl. Food intake was measured daily. Body and fasting blood glucose measurements were recorded, and then the mice were sacrificed. Blood and liver samples were harvested after the injection period. Blood was drawn by cardiac puncture. All experimental protocols were approved by the Animal Experimentation Ethics Committee of Shanghai Jiao Tong University Affiliated Sixth People’s Hospital in accordance with the guidelines of the Institutional Animal Care and Use Committee (IACUC) of Shanghai Jiao Tong University.

### Biochemical Examinations and Enzyme-Linked Immunosorbent Assays (ELISAs)

Serum and hepatic levels of total cholesterol (TC) and triglyceride (TG) were determined using commercially available kits provided by Nanjing Jiancheng Institute of Biotechnology (Nanjing, China), according to the manufacturer’s protocol. The extraction and analysis of liver lipids were conducted according to the procedure described by Kim et al. (2017)’s Materials and Procedure—extended for details, http://dx.doi.org/10.17504/protocols.io.iy7cfzn. Serum insulin and serum and hepatic IL-6 were measured using a mouse insulin ELISA kit (Alpine Immune Sciences, Hong Kong, China) and a mouse IL-6 ELISA kit (Biovendor, Czech Republic), respectively, following the manufacturer’s instructions.

### Assay of Antioxidant Markers

The liver specimens were homogenized in PBS (phosphate-buffered saline) to prepare a 10% liver homogenate, and centrifugated at 3,000rpm for 10 min at 4°C. The supernatant was then collected for the assays. Hepatic malondialdehyde (MDA) activities, superoxide dismutase (SOD) levels, and glutathione (GSH) content were measured spectrophotometrically using commercially available kits supplied by the Nanjing Jiancheng Institute of Biotechnology (Nanjing, China). All assays were performed according to the manufacturer’s instructions.

### Western Blot Analysis

Western blot was performed using a standard protocol. Tissue specimens were homogenized and lysed with RIPA lysis buffer. The concentrations of total protein were determined using the BCA (bicinchoninic acid) method. Proteins were separated equally from each specimen by sodium dodecyl sulfate-polyacrylamide gel electrophoresis (SDS-PAGE) and transferred onto polyvinylidene fluoride (PVDF) membranes. Thereafter, the membranes were blocked with 5% skim milk and incubated overnight with the appropriate antibodies at 4°C followed by horseradish peroxidase (HRP)-labeled secondary antibody for 1 h at room temperature. The primary antibodies included GSK-3β rabbit mAb (1:1,000; Cell Signaling Technology Inc., the US. #12456), phospho-GSK-3β (Ser9) rabbit mAb (1:1,000; Cell Signaling Technology Inc. #5558), mTOR rabbit mAb (1:1,000; Cell Signaling Technology Inc. #2983), phospho-mTOR (Ser2448) rabbit mAb (1:1,000; Cell Signaling Technology Inc. #5536), AKT rabbit mAb (1:1,000; Cell Signaling Technology Inc. #4691), phospho-AKT (Ser473) rabbit mAb (1:1,000; Cell Signaling Technology Inc. #4060), P53 rabbit mAb (1:1,000; Proteintech #10442-1-AP), P16 rabbit mAb (1:1,000; abcam.ab51243), β-gal rabbit mAb (1:1,000; Cell Signaling Technology Inc. #27198), LC3I/II rabbit mAb (1:1,000; Cell Signaling Technology Inc. #3868), P62 rabbit mAb (1:1,000; Cell Signaling Technology Inc. #88588), beclin-1 rabbit mAb (1:1,000; Cell Signaling Technology Inc. #3495), and GAPDH rabbit mAb (1:5,000; abcam.ab8245). Finally, the immunoreacting bands were visualized using an enhanced chemiluminescence (ECL) method.

### Oil Red O Staining

Frozen specimens were cut into thin sections about 10 μm thick and then stained with filtered Oil Red O (cat. no. 1.02419; EMD Millipore, Billerica, MA, USA) dissolved in 60% isopropanol for 15 min at room temperature. Next, the slides were incubated in hematoxylin to counterstain the nuclei and transferred to aqueous mounting medium. Images were captured under a light microscope (magnification ×200 and ×400).

### Transmission Electron Microscopy

Liver samples were fixed in 3% glutaraldehyde for 24 h at 4°C, post-fixed in 1% osmium tetroxide in sodium phosphate buffer at room temperature, and cut into ultrathin sections (50–70 nm) on an ultramicrotome. Sections were stained with 2% uranyl acetate and lead citrate, and then viewed and photographed using a Hitachi H-7650 transmission electron microscope at 120 kV. Ultrastructural analysis was performed using ImageJ (NIH) analysis.

### Statistical Analysis

Sample sizes, as described in the figure legends, were selected based on effect size and availability, according to the usual standard. Statistical analysis was performed using GraphPad Prism 7.0 software. Data were presented as mean ± standard deviation (SD). One-way ANOVA analysis was applied to assess the statistical significance, using Tukey’s *post hoc* analysis when required. *p* < 0.05 was considered statistically significant.

## Results

### 6BIO Attenuated Hepatic Oxidative Stress and Inflammation

**Figure 1:**
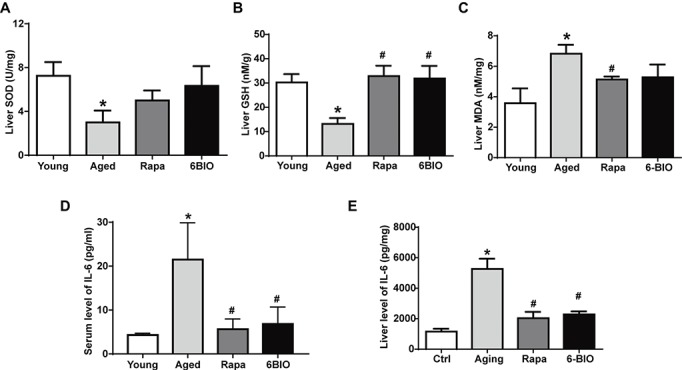
6BIO attenuated hepatic oxidative stress and inflammation. The levels of **(A)** SOD, **(B)** GSH, and **(C)** MDA in liver tissue were measured by using commercially available kits. The **(D)** serum and **(E)** hepatic levels of IL-6 were measured by ELISAs. The results are shown as the mean ± SD of eight animals per group. *p < 0.05 compared with the young control group; ^#^ *p* < 0.05 compared with the aged control group; ^&^ *p* < 0.05 compared with the rapamycin treatment group.

Oxidative stress is strongly believed to contribute to the aging and the pathogenesis of a considerable number of degenerative diseases (Yew et al., 2018). Therefore, we evaluated the effects of 6BIO on aged mouse liver in relation to antioxidant enzymes SOD, GSH, and MDA. As shown in Figures 1A–C, compared with the young mouse liver, in the aged mouse liver, the level of SOD and GSH was significantly (*p* < 0.05) reduced, while the level of MDA was significantly (*p* < 0.05) increased. The treatment with 6BIO or rapamycin was observed to bring the level of SOD close to that in the young control. 6BIO and rapamycin significantly (*p* < 0.05) increased the level of GSH in the old mouse liver. In addition, they reduced the level of MDA.

Aging is also accompanied by increased levels of pro-inflammatory cytokines such as IL-6; this appears to be linked to the immunosenescence process (da Cunha and Arruda, 2017). As shown in Figures 1D,E, the mean values of both serum and hepatic IL-6 were significantly (*p* < 0.05) higher in the aged group than in the young control group. The 6BIO-treated group and rapamycin-treated group showed significantly (*p* < 0.05) lower IL-6 levels in serum and liver than the untreated aged group.

These results suggest that 6BIO and rapamycin treatment may attenuate oxidative stress and inflammation associated with aging in rodent liver.

### 2-Week Treatment With 6BIO, Compared With Rapamycin, Exerted a Strong Effect on Hepatic Lipid Metabolism but Only a Mild Effect on Glucose Metabolism

**Figure 2:**
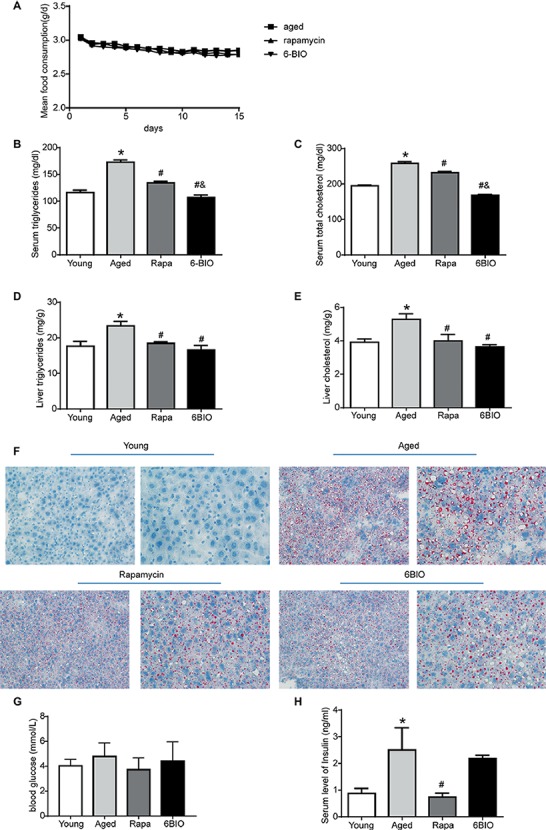
Two-week treatment with 6BIO, compared with rapamycin, exerted a strong effect on hepatic lipid metabolism, but little effect on glucose metabolism. **(A)** Mean food consumption per mouse per day **(B)** Serum triglycerides. **(C)** Serum total cholesterol. **(D)** Liver triglycerides. **(E)** Liver cholesterol. **(F)** Liver steatosis assessed by Oil Red O staining. **(G)** Blood glucose. **(H)** Serum level of insulin. All values were expressed as mean ± SD of eight animals per group. *p < 0.05 compared with the young control group; ^#^ *p* < 0.05 compared with the aged control group; ^&^ *p* < 0.05 compared with the rapamycin treatment group.

There was no significant difference in mean food intake between the aging mice, the rapamycin-treated mice, and the 6BIO-treated mice (Figure 2A). The changes in serum lipid levels are shown in Figures 2B,C. Analyses of serum lipid concentration showed significantly (*p* < 0.05) higher levels of serum triglycerides (TG) and TC in the aged group than in the young control group. 6BIO and rapamycin significantly (*p* < 0.05) reduced the average levels of both serum TG and serum TC in the aged mice. The serum lipid-reducing effect of 6BIO was even significantly (*p* < 0.05) stronger than that of rapamycin.

We next analyzed the levels of hepatic TG and TC content (Figures 2D,E). 6BIO and rapamycin significantly (*p* < 0.05) reduced the average levels of hepatic TG and TC in the old-aged mice, closer to the level observed in the young control group. To further investigate whether 6BIO was able to affect hepatic steatosis, we then performed Oil Red O staining to illustrate the hepatic lipid content (Figure 2F). Histological analysis of the liver specimens showed marked hepatic steatosis in the old-aged group. A remarkably decreased accumulation of lipid droplets in the aged liver was observed in both the 6BIO-treated group and the rapamycin-treated group.

We also investigated the effect of 6BIO on glucose metabolism in the mice. Accordingly, the 2-week treatment with rapamycin or 6-BIO did not significantly alter blood glucose levels (Figure 2G). Nevertheless, rapamycin significantly (*p* < 0.05) reduced the level of fasting insulin, thereby indicating an improvement in insulin sensitivity. In the 6BIO-treated group, however, no significant difference was observed in fasting insulin compared to the aged control group (Figure 2H).

Overall, 6BIO reduced serum lipid concentration and lipid accumulation in liver and ameliorated the hepatic steatosis. The treatment with 6BIO even showed a mildly stronger effect than the rapamycin treatment on lipid metabolism. Nevertheless, the 2-week 6BIO treatment resulted in little improvement in insulin sensitivity.

### 6BIO Regulated the Senescence Markers of Mice Livers

To verify the effects of 6BIO on hepatic senescence, Western blot was used to analyze the levels of the senescence markers p16, p53, and β-gal (β-galactosidase). Previous studies had reported the upregulation of p16 and β-gal and the downregulation of p53 to be associated with age-related dysfunction. These molecular markers of senescence could be promoted as well-established endpoints when investigating anti-aging interventions (Niedernhofer et al., 2017).

**Figure 3:**
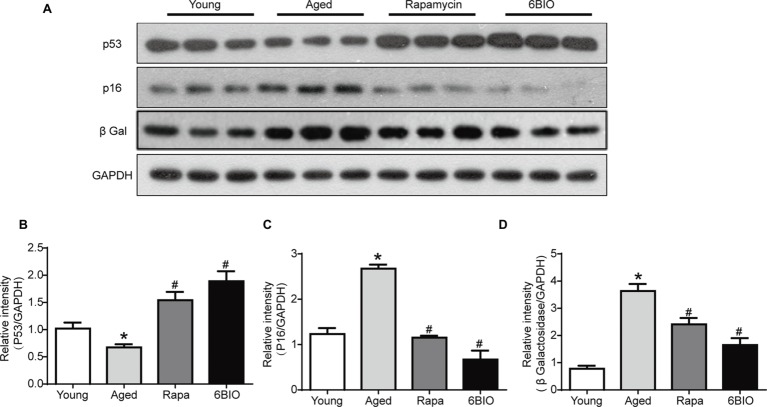
6BIO regulated the senescence markers of mouse livers. **(A)** Western blotting results revealed the changes in senescence markers. Protein expression levels of **(B)** p53, **(C)** p16, and **(D)** β-gal were evaluated by Western blot analysis. The results are shown as the mean ± SD of eight animals per group. *p < 0.05 compared with the young control group; ^#^ *p* < 0.05 compared with the aged control group; ^&^ *p* < 0.05 compared with the rapamycin treatment group. GADPH was used as a loading control.

As shown in Figures 3A–D, compared with the young control group, in the aged mouse liver, the levels of p16 and β-gal were found to be significantly elevated, while the expression of p53 decreased markedly, in line with the findings of other studies (Roos et al., 2016). The expression of p53 in both the 6BIO-treated group and the rapamycin-treated group was higher than that in the untreated old group (*p* < 0.05), while the levels of p16 and β-gal were significantly elevated in both the 6BIO-treated group and rapamycin-treated group.

Overall, both 6BIO and rapamycin exhibited a marked anti-aging effect. 6BIO treatment even showed a slightly better anti-aging effect than the rapamycin treatment, which is deemed to be one of the most promising anti-aging drugs (Wang et al., 2017).

### 6BIO Activated Autophagy in Old-Aged Murine Livers

**Figure 4:**
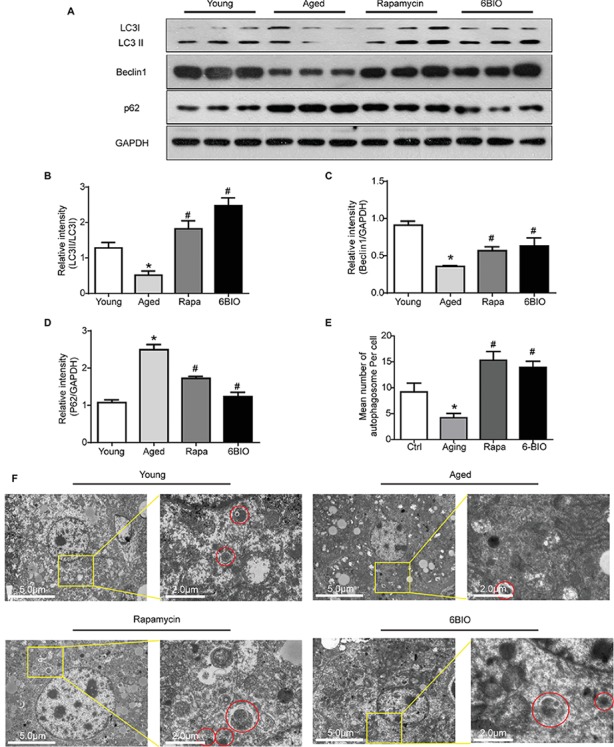
6BIO activated autophagy in aged murine livers. **(A)** Western blotting and the analysis of the Western blot results of autophagy-related proteins **(B)** LC3II/I, **(C)** beclin1, and **(D)** p62. **(E)** Mean number of autophagosome per cell under electron microscope (three hepatocytes were counted per group at 1.2k× magnification). **(F)** Observation of autophagosomes through an electron microscope. All values were expressed as mean ± SD of eight animals per group. *p < 0.05 compared with the young control group; ^#^ *p* < 0.05 compared with the aged control group; ^&^ *p* < 0.05 compared with the rapamycin treatment group.

It has been widely reported that autophagy plays a crucial role in the progression of aging (Nakamura and Yoshimori, 2018). Hence, we investigated whether autophagy is involved in the anti-aging effect of 6BIO. As shown in Figures 4A–D, the expression level of autophagy-related proteins LC3, Beclin1, and p62 was measured by Western blot. 6BIO treatment, as with rapamycin treatment, significantly increased the LC3II:LC3I ratio and the level of Beclin1, along with markedly reducing the level of p62 in the aged mouse liver. In addition, under the transmission electron microscope, fewer autophagosomes were observed in the aged mouse liver than in the young mouse liver, whereas more autophagosomes were observed in the liver specimen of the 6BIO-treated group and the rapamycin-treated group (Figures 4E,F).

Taken together, these results indicate that 6BIO is a promising autophagy inducer, and accordingly, autophagy may play a pivotal role in the positive effect of 6BIO treatment.

### 6BIO Inhibited Both the mTOR and the GSK-3β Signaling Pathway

**Figure 5:**
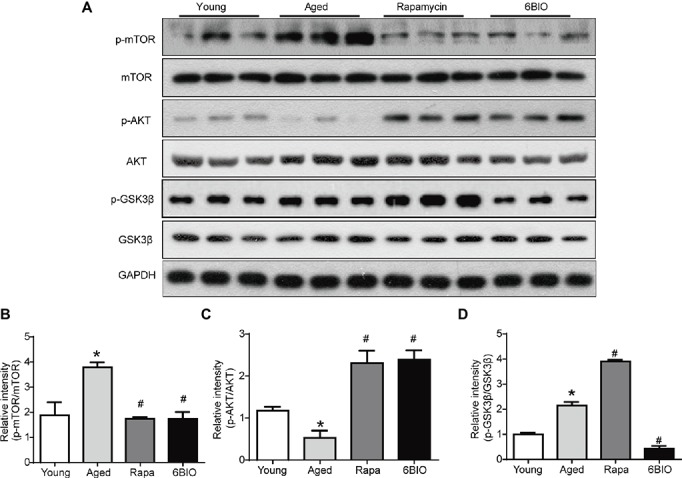
6BIO inhibited both the mTOR and the GSK-3β signaling pathway. **(A)** The phosphorylated mTOR, the total mTOR expression, the phosphorylated Akt, the total Akt expression, the phosphorylated GSK-3β, and the total GSK-3β expression were examined by Western blot. **(B)** p-mTOR to mTOR ratio. **(C)** p-Akt to Akt ratio. **(D)** p-GSK-3β to GSK-3β ratio. The results are shown as the mean ± SD of eight animals per group. *p < 0.05 compared with the young control group; ^#^ *p* < 0.05 compared with the aged control group; ^&^ *p* < 0.05 compared with the rapamycin treatment group.

It has been reported that the mTOR signaling pathway plays a pivotal role in the process of aging (Baar et al., 2016), and that it is a critical mechanism in terms of regulating autophagic capacity (Wong et al., 2017). To confirm the mechanism of the 6BIO-induced anti-aging effect, we investigated the mTOR signaling pathway. According to Western blotting analysis, both the 6BIO treatment and the rapamycin treatment inhibited phosphorylation of the mTOR (Figures 5A,B). In both the 6BIO-treated group and the rapamycin-treated group, Akt phosphorylation was significantly higher than that in the aged untreated group (Figures 5A,C), which supports the hypothesis that due to the inhibition of mTOR, the reduction in its downstream molecule S6K attenuates the S6K-mediated negative feedback control, thereby resulting in Akt activation (Lashinger et al., 2011).

As 6BIO is a known potent inhibitor of GSK-3α/β, we then explored the expression of p-GSK-3β. The level of GSK-3β phosphorylation on serine-9 was reduced significantly in the 6BIO-treated group, while conversely, the rapamycin-treated group exhibited an increasing level of p-Gsk-3β with increasing upregulation of Akt phosphorylation compared with the aged group (Figures 5A,C,D). GSK-3β Serine-9 phosphorylation is part of a negative feedback loop and 6BIO-mediated inhibition of GSK-3B activity was previously shown to be associated with the downregulation of this inhibitory phosphorylation (Sklirou et al., 2017).

These results indicate that 6BIO inhibits both the mTOR and the GSK-3β signaling pathway, while rapamycin positively regulates GSK-3β signaling.

## Discussion

Anti-aging pharmacology helps prevent the vast majority of degenerative disorders and prolong lifespan by delaying aging rather than targeting all the age-related pathological manifestations one by one (Kennedy and Pennypacker, 2014). It has been reported that 6BIO, a potent inhibitor of GSK-3α/β, has made a promising impact on age-associated diseases such as cancer and neurodegenerative diseases (Beurel et al., 2015; McCubrey et al., 2016). By contrast, little research has focused on the effect, and mechanism of action, of this potent drug on models of natural aging, especially liver aging. In this study, we aimed to investigate the anti-aging effect, and molecular mechanism, of the novel anti-aging drug 6BIO on naturally aged mouse liver. Rapamycin, a well-known promising anti-aging drug that delays aging through mTOR-dependent autophagy (Zhou and Ye, 2018), was used as the positive control in the study. To our knowledge, this is the first study to demonstrate the effects of 6BIO treatment in models of natural aging.

Our results indicated that 6BIO ameliorates the decline of liver function with age, including lipid metabolism disorder, and attenuates hepatocyte senescence in aged mice, as revealed by alterations in the cellular senescence markers. Also, we investigated the possible molecular anti-aging mechanisms of action, and respective signaling pathways, of 6BIO, some of which relate to age-associated changes, too, such as the changes in canonical p53/p21 and p16/pRB signaling pathways, oxidative stress, inflammation, autophagy, the GSK-3β signaling pathway, and the mTOR signaling pathway.

Lipid and glucose metabolic disorders are common manifestations in aging models, thereby correlating closely with a decline in liver function. One characteristic of hepatic senescence is fat deposition (He et al., 2018). Our results showed an increase in fat deposition and disordered lipid metabolism during the aging process of the aged mice. In the 6BIO treatment group, the serum levels of TC and TG decreased, thereby indicating an amelioration of lipid metabolic disorders as a result of improved liver function. Moreover, 6BIO treatment rectifies the hepatic levels of TC and TG, and lipid droplet accumulation in liver. Overall, the 6BIO treatment even exerted a slightly better effect on lipid metabolism—or, in other words, on lipid metabolism in liver—than the rapamycin treatment. Apart from lipid-related metabolic disorder, the higher serum level of insulin in the aged group suggested an impaired insulin sensitivity resulting from aging. Nevertheless, a 2-week treatment with 6BIO may be too short a duration to exert a significant improvement in insulin sensitivity.

We also examined cell senescence markers to better understand aging in hepatocytes. Molecular senescence markers such as p53, p16, and β-gal are well-established endpoints for quantifying the degree of cell aging (Niedernhofer et al., 2017). According to our findings and those of other studies, p53 was downregulated, and the level of p16 elevated, by the aging process in the aged mice. β-Gal is an enzyme whose protein expression levels in the aged mice were found to be higher than in the young control mice in our study, which is consistent with the findings of another study (Niedernhofer et al., 2017). 6BIO treatment significantly ameliorated the level of these senescence markers, indicating the attenuation of cell senescence. Moreover, p53 and p16 are not only the biomarkers of cell senescence but also controllable factors regulating the aging process (Yi et al., 2013). It is reported that p53 extends lifespan and maintains normal tissue homeostasis (Qian and Chen, 2013), the ablation of p16 cells in rodents promotes longevity and alleviates age-related dysfunction (Baker et al., 2016), the deficiency of p16 induces elevated p53, and the activity of β-gal is strongly associated with the expression of p16 (Niedernhofer et al., 2017). Hence, our finding that 6BIO activated p53, reduced p16, and diminished β-gal also suggests that an overexpression of p53, reduction in p16, and downregulation of β-gal could constitute a possible pathway for the anti-aging effect of 6BIO. Further research is needed to determine the specific interactions between 6BIO and these molecules.

Oxidative stress and inflammation are two of the most well-known canonical aging mechanisms (Park et al., 2014; Zhang et al., 2015). SOD functions as an antioxidant enzyme, while GSH is a most vital catalase and a nonenzymatic antioxidant. The end product of lipid peroxidation, MDA, is measured to reflect the level of hepatic oxidative damage. Similar to the findings of previous studies (Jimenez et al., 2018), the aged mice exhibited lower levels of SOD and GSH than the young mice, whereas a marked increase in MDA was observed in the aged group, indicating that liver oxidative stress increases with increasing age. Studies also report that the production of pro-inflammatory cytokines, such as IL-6, is linked to the aging process (Kim et al., 2016). Our results showed that the aging process was accompanied by a hepatic pro-inflammatory response, which is reflected by the upregulation of IL-6. In the 6BIO treatment group, a marked improvement in oxidative stress and inflammation in the aged livers was observed in our study. SOD and GSH activities were found to be significantly elevated, MDA levels decreased markedly, and the IL-6 expression was significantly upregulated when the aged mice were treated with 6BIO. Oxidative stress and inflammation were found to be similarly diminished in the rapamycin treatment group.

Autophagy is a vital degradation process that controls cellular and organismal homeostasis in mammals and plays a pivotal role in liver aging. There is a strong correlation between autophagy and age-associated liver dysfunction (Czaja, 2010). In addition, recent studies have revealed that 6BIO is a novel autophagy inducer in human diploid skin fibroblasts and in dopaminergic neurons of mice midbrain. Nevertheless, so far, no research has proven whether this agent regulates autophagy in liver. In the present study, the decrease in LC3II:I ratio and beclin-1 level, the increase in p62 level, and the reduced number of autophagosomes observed under microscopy in the aged group demonstrated a decline in autophagy with aging. Our findings showed that 6-BIO remarkably induces autophagy. Our study revealed—for the first time—that 6BIO treatment induces autophagy in aging liver. 6BIO may delay aging and ameliorate age-related alterations through an autophagy pathway.

**Figure 6:**
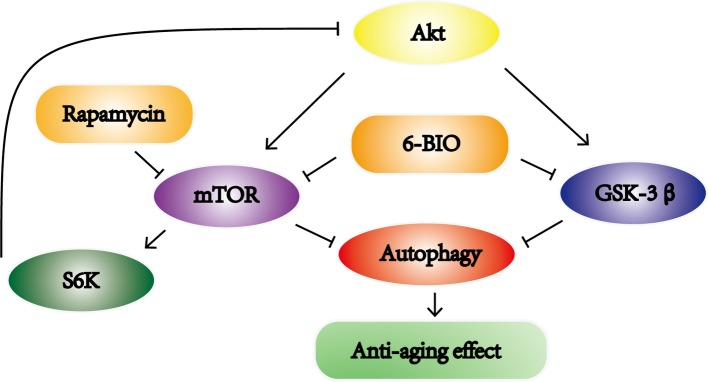
Schematic illustration of this study. 6BIO treatment induced autophagy to exert an anti-aging effect by inhibiting mTOR and GSK-3β signaling.

Meanwhile, taking into consideration that the mTOR pathway negatively controls autophagy, we also investigated whether 6-BIO affects the mTOR signaling pathway. Since 6BIO is known to be a potent GSK-3α/β inhibitor, it seemed logical to observe the GSK-3β pathway also. The level of Akt phosphorylation was observed because Akt is upstream of both the mTOR pathway and GSK-3β pathway. Previous studies have reported that inhibition of the mTOR pathway and GSK-3β pathway ameliorated age-related pathologic changes (Chen et al., 2018; Yuan et al., 2018). The results showed that 6BIO not only inhibits GSK-3β signaling, in concordance with the findings of previous studies (Ko et al., 2018), but also inhibits mTOR signaling, whereas rapamycin inhibits the mTOR pathway but activates GSK-3β signaling *via* feedback activation of Akt originating from S6K (Wu et al., 2018). Moreover, the phosphatidylinositol-3 kinase (PI3-K)/phosphoinositide-dependent protein kinase 1 (PDK1)/Akt pathway, which positively regulates cell growth, is implicated in some anti-aging mechanisms (Haga et al., 2009). The inhibition of PI3K or PDK1 will lead to the suppression of Akt phosphorylation and downstream mTOR signaling. However, the increase in the p-Akt/Akt ratio in the 6BIO-treated group indicates that 6BIO inhibits the phosphorylation of mTOR and GSK-3β rather than affecting PDK1/Akt signaling. In previous studies, 6BIO was found to alleviate age-related diseases *via* the GSK-3β pathway. Our own results indicated that 6BIO may exert its anti-aging effect on liver *via* crosstalk with another novel pathway—the mTOR signaling pathway. It is reported that, additionally, GSK-3β inhibition may induce autophagy in human pancreatic cancer cells and in prostate cancer cells (Marchand et al., 2015; Sun et al., 2016). Overall, our results support the model illustrated in Figure 6. 6BIO treatment might induce autophagy to exert an anti-aging effect *via* the inhibition of both mTOR and GSK-3β signaling.

Currently, 6BIO has been tested in certain preclinical disease models, such as in Parkinson disease models, intracerebral hemorrhage models, and leukemia models, and such research has provided promising evidence of efficacy (Shen et al., 2015; Suresh et al., 2017; Zhao et al., 2017). Our study also points to the clinical potential of therapeutic 6BIO in liver. However, to date, no clinical trial concerning 6BIO has been conducted. In fact, some matters still require attention before being applied to a clinical setting. A recent study indicated that GSK-3β inhibitor suppresses B- and T-cell development in stem cells, which signifies it should be tested carefully, particularly in conjunction with stem cell transplantation and adoptive T-cell immunotherapy (Shen et al., 2015).

In summary, according to this study, 6BIO delays liver aging and ameliorates age-associated alterations, including lipid metabolism, hepatic oxidative stress, and hepatic inflammation. In particular, in terms of balancing lipid metabolism, 6BIO seems to be even slightly more effective than rapamycin. Besides, the levels of the senescence markers also indicated that the 6BIO-treated group fared better, although not significantly, than the rapamycin-treated group. Overall, despite the fact that rapamycin appears to have a more positive effect than 6BIO on insulin resistance, the 6BIO treatment showed a remarkable anti-aging effect, functioning even slightly better than the rapamycin treatment, which is deemed to be one of the most promising anti-aging drugs (Wang et al., 2017). Accordingly, we suggest that besides inhibiting the GSK-3β pathway, 6BIO may exert its anti-aging effect in liver by inducing autophagy through mTOR inhibition. FOXO transcription factors are important mediators of autophagy-related gene expression and aging (Martins et al., 2016). It will be interesting to analyze how these factors are regulated in liver upon 6BIO-treatment. Cytoprotective effects of 6BIO were shown to depend on Nrf2, an inducer of antioxidant genes which is compromised during aging (Sklirou et al., 2017; Tsakiri et al., 2017; Zhou et al., 2018). Understanding the exact nature of the underlying mechanisms by which 6BIO delays or reverses aging downstream to mTOR/GSK-3B requires further investigations possibly in these directions.

## Author Contributions

DG designed the research, performed the experiments, analyzed the data, and drafted the manuscript. YZho and YM supervised the research, critically revised the manuscript for important intellectual content, and obtained funding. YS, WL, QL, YZha, CP, and BC were also involved in performing the experiments. All authors read and approved the final manuscript.

### Conflict of Interest Statement

The authors declare that the research was conducted in the absence of any commercial or financial relationships that could be construed as a potential conflict of interest.

The handling editor and reviewers TZ and NE declared their involvement as co-editors in the Research Topic, and confirm the absence of any other collaboration.
